# Single-Centre Retrospective Study Using Propensity Score Matching Comparing Left Versus Right Internal Jugular Vein Access for Transjugular Intrahepatic Portosystemic Shunt (TIPS) Creation

**DOI:** 10.1007/s00270-021-03023-9

**Published:** 2022-01-01

**Authors:** Zhenkang Qiu, Wenliang Zhu, Huzheng Yan, Guobao Wang, Mengxuan Zuo, Han Qi, Guisong Wang, Weiwei Jiang, Jingbing Xue, Fujun Zhang, Fei Gao

**Affiliations:** 1 Department of Minimally Invasive & Interventional Radiology, Sun Yat-Sen University Cancer Center and Sun Yat-Sen University State Key Laboratory of Oncology in South China, Collaborative Innovation Center for Cancer Medicine, 651 Dongfeng East Road, Guangdong, 510060 Guangdong Province China; 2 Department of Interventional Radiology, The Third Affiliated Hospital of Sun Yat-Sen University, Guangzhou, China; 3 Department of Endoscopy, Collaborative Innovation Center for Cancer Medicine, Sun Yat-Sen University Cancer Center and Sun Yat-Sen University State Key Laboratory of Oncology in South China, Guangzhou, Guangdong China; 4 Department of Imaging Sciences, University of Rochester Medical Center, Rochester, NY USA

**Keywords:** TIPS, Left internal jugular vein access, Liver cirrhosis

## Abstract

**Purpose:**

To compare the safety and efficacy of left versus right internal jugular vein access for portal vein puncture during transjugular intrahepatic portosystemic shunt (TIPS) creation in patients with a small liver and short vertical puncture distance.

**Materials and Methods:**

The vertical distance from the hepatic vein orifice to the puncture point of the portal vein was measured by CT and DSA. A distance ≤ 30 mm is defined as a short vertical puncture distance. After 1:1 propensity score matching (PSM), 29 patients of left internal jugular vein-TIPS (LIJ-TIPS) and 29 patients of right internal jugular vein-TIPS (RIJ-TIPS) were included. The number of needle punctures, fluoroscopy time, and radiation dose during the puncture process were statistically analyzed.

**Results:**

There was no significant difference in the average vertical puncture distances on CT or DSA between LIJ-TIPS and RIJ-TIPS (19.10 ± 0.60 mm *vs.* 19.30 ± 0.60 mm, *P* = 0.840; 22.02 ± 0.69 mm *vs.* 22.23 ± 0.64 mm, *P* = 0.822, respectively). The average number of needle punctures, fluoroscopy time, and radiation dose in LIJ-TIPS were significantly lower than those in RIJ-TIPS (2.07 ± 0.20 *vs.* 4.10 ± 0.24, *P* < 0.001; 78.45 ± 12.80 s *vs.* 201.16 ± 23.71 s, *P* < 0.001; 31.55 ± 7.04 mGy *vs.* 136.69 ± 16.38 mGy, *P* < 0.001, respectively). Within three punctures, the technical success rate in LIJ-TIPS was significantly higher than that in RIJ-TIPS (86.2 *vs.* 27.6%, *P* < 0.001). The incidence of hemoperitoneum in LIJ-TIPS was significantly lower than that in RIJ-TIPS (0% *vs.* 13.8%, *P* = 0.038).

**Conclusion:**

The left internal jugular vein could be used as primary access for TIPS creation in patients with a small liver and short vertical puncture distance.

**Supplementary Information:**

The online version contains supplementary material available at 10.1007/s00270-021-03023-9.

## Introduction

Transjugular intrahepatic portosystemic shunt (TIPS) has resulted in significant progress in the management of portal hypertension-related complications in patients with cirrhosis [1–4]. More than 90% of TIPS procedures in experienced centers can be performed via the right internal jugular vein (RIJ), a preferred access for the procedure [5, 6]. The main characteristic of severe cirrhosis is a reduction in liver volume, which shortens the distance between the hepatic vein and the portal vein [7, 8]. In clinical practice, the left internal jugular vein (LIJ) can be used for a second TIPS attempt when the RIJ is not available for access [9, 10]. However, limited data comparing RIJ versus LIJ access for TIPS creation exist. This study compared LIJ versus RIJ access for TIPS creation.

## Materials and Methods

**Fig. 1:**
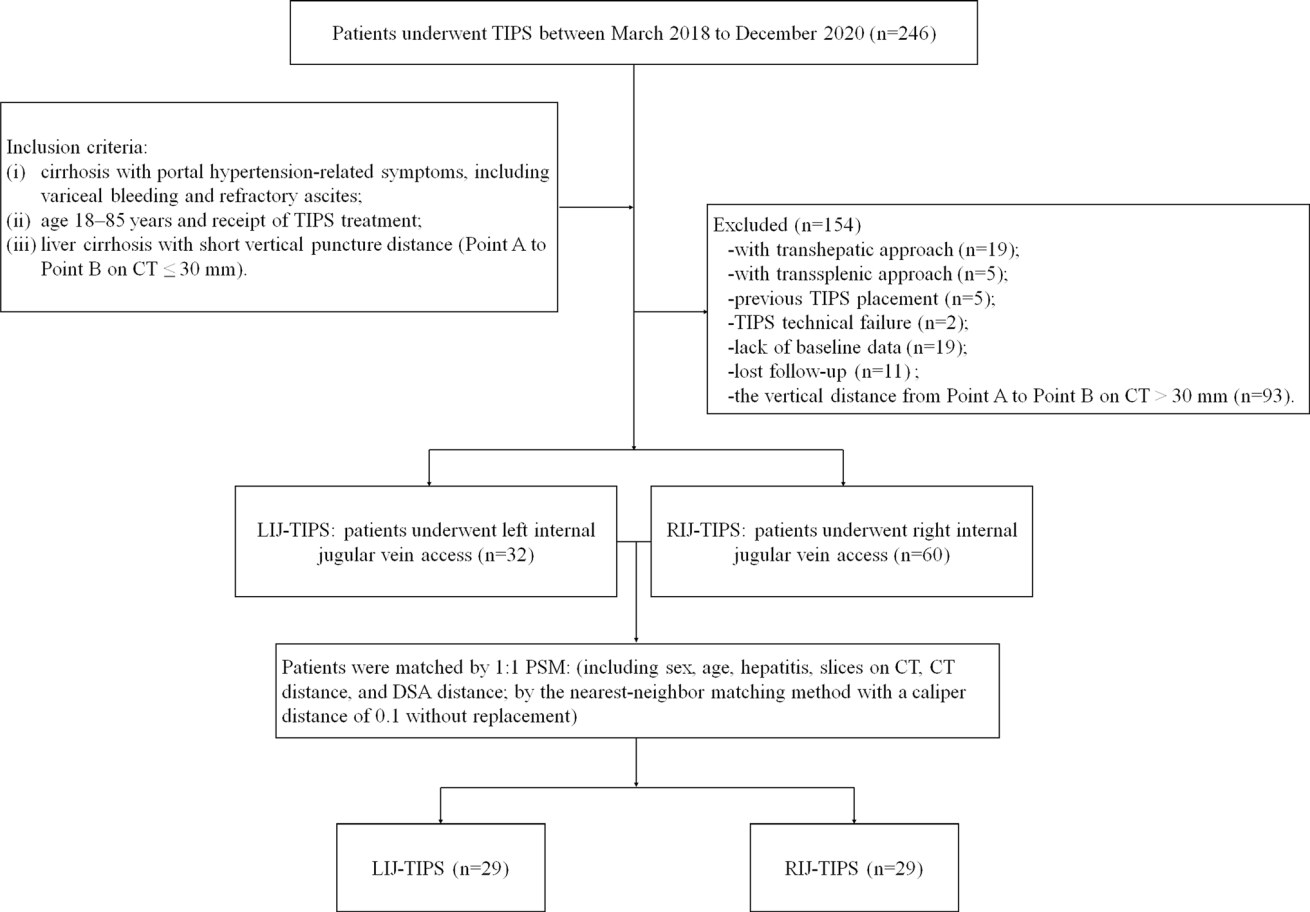
Patient flow diagram. The inclusion criteria were as follows: (i) cirrhosis with portal hypertension-related symptoms, including variceal bleeding and refractory ascites; (ii) age 18–85 years and receipt of TIPS treatment; (iii) liver cirrhosis with short vertical puncture distance (Point A to Point B on CT ≤ 30 mm). Patients with transhepatic approach (n = 19); with transsplenic approach (*n* = 5); previous TIPS placement (*n* = 5); TIPS technical failure because of portal vein occlusion and cavernous degeneration (*n* = 2); lack of baseline data (*n* = 19); lost follow-up (*n* = 11); and the vertical distance from Point A to Point B on CT > 30 mm (*n* = 93) were excluded from the study. Note: The vertical distance from Point A to Point B is the distance of vertical slices (0.5 cm thick) from the hepatic vein orifice to the puncture point of the portal vein on preoperative CT. LIJ access was used after a failed TIPS attempt from the RIJ access (*n* = 10). LIJ was chosen as first-line access upfront because of the short vertical puncture distance (*n* = 19). TIPS, transjugular intrahepatic portosystemic shunt; LIJ-TIPS, left internal jugular vein access; RIJ-TIPS, right internal jugular vein access; PSM, propensity score matching

This single-center retrospective study was performed according to the Declaration of Helsinki (2013) of the World Medical Association and approved by the Institutional Review Board of our center, who waived the need for informed patient consent. The vertical puncture distance on CT was converted with the distance of vertical slices (0.5 cm thick) from the hepatic vein orifice (Point A) to the puncture point of the portal vein (Point B) on preoperative CT. The vertical puncture distance on digital subtraction angiography (DSA) images was confirmed and measured directly with the computer's Picture Archiving and Communication Systems (PACS). A distance ≤ 30 mm is defined as a short vertical puncture distance. Patients were divided into the LIJ-TIPS group or the RIJ-TIPS group according to TIPS access; ultimately, a total of 29 patients were included in each group (Fig. 1).

**Fig. 2:**
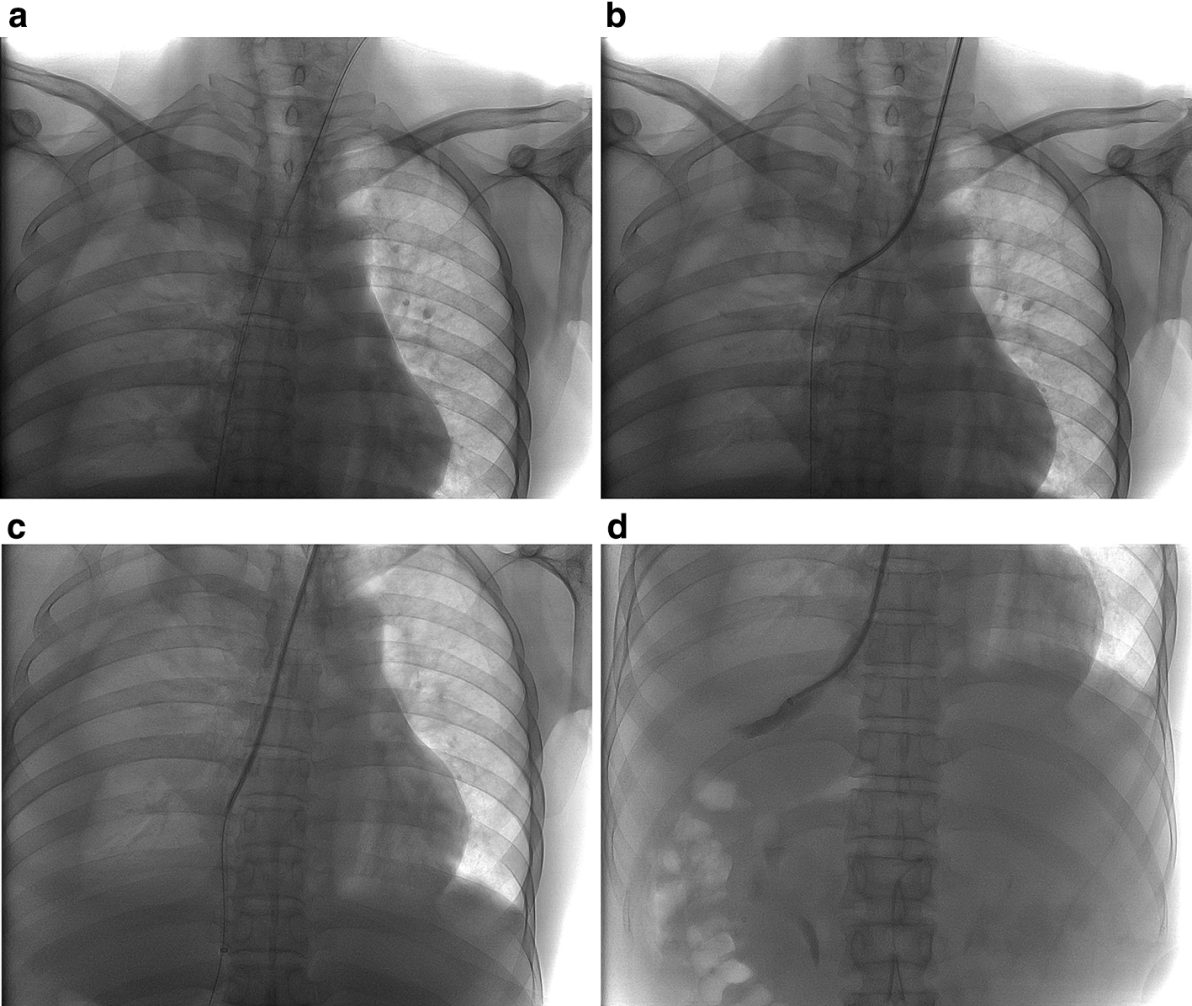
Entrance of the stiffening cannula into the middle hepatic vein through the left internal jugular vein. **a** A 10-Fr sheath is straightening the superior vena cava. **b** The stiffening cannula enters into the superior vena cava. **c** The stiffening cannula is passing through the right atrium. **d** Middle hepatic venography

**Fig. 3:**
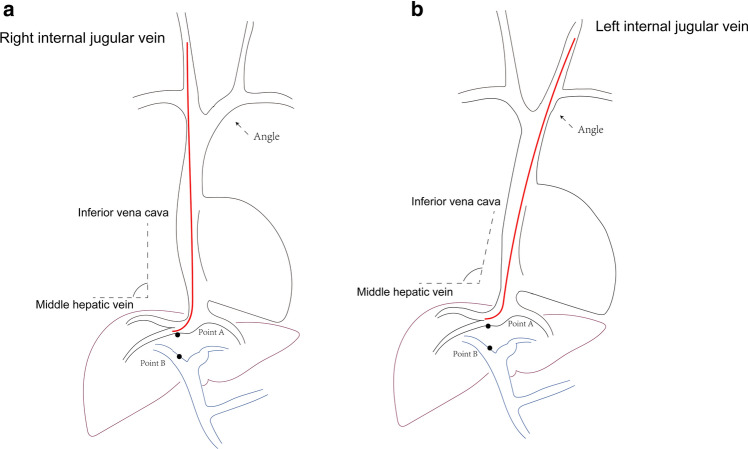
Schematic diagram of RIJ-TIPS and LIJ-TIPS. **a** TIPS with right internal jugular vein access (RIJ-TIPS). **b** TIPS with left internal jugular vein access (LIJ-TIPS). Point A, hepatic vein orifice; Point B, puncture point of the portal vein; TIPS, transjugular intrahepatic portosystemic shunt

**Fig. 4:**
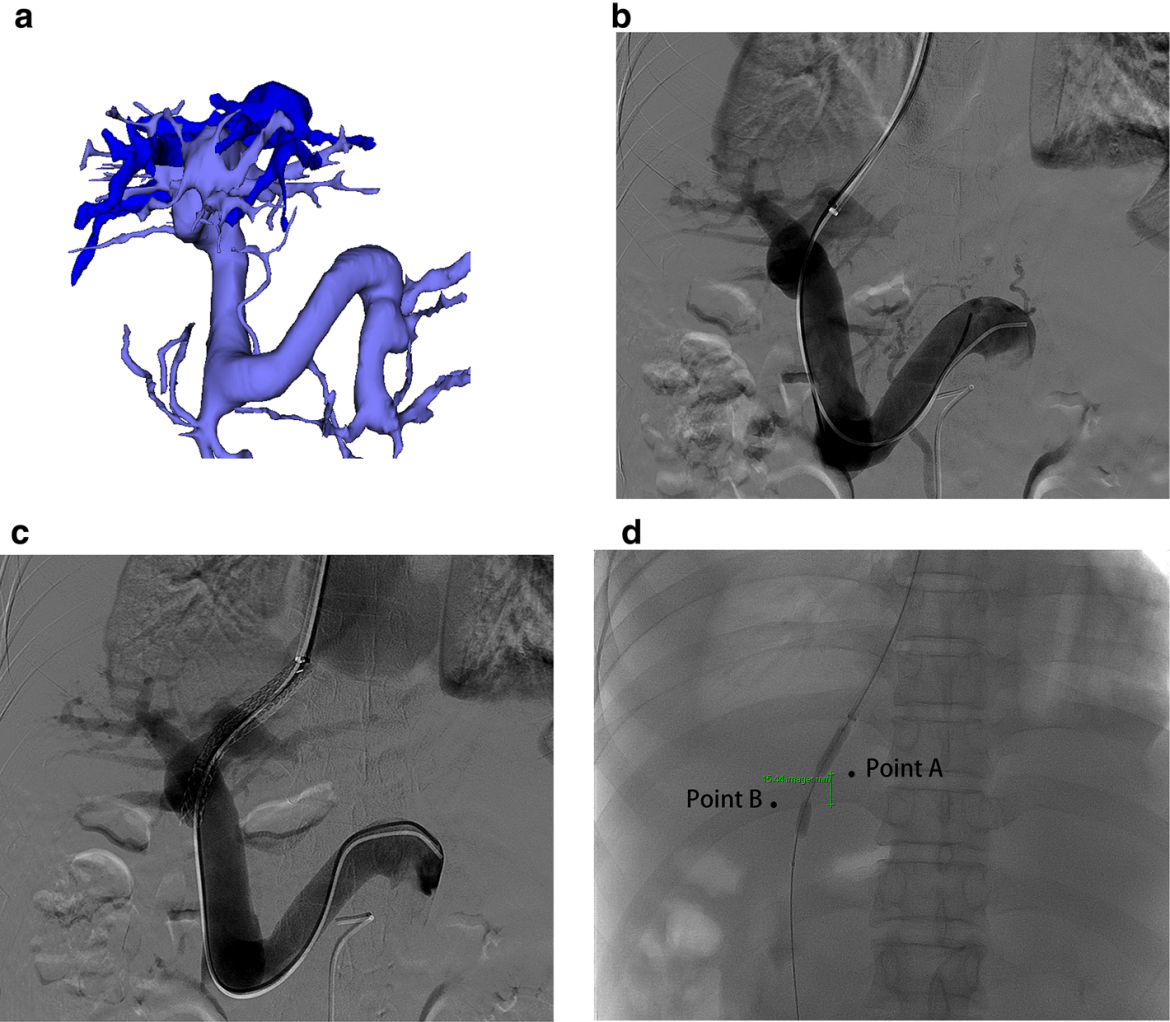
A 42-year-old male patient suffering from refractory ascites received a transjugular intrahepatic portosystemic shunt (TIPS) with left internal jugular vein access. **a** An anteroposterior 3D image shows the short vertical distance between the hepatic vein and the portal vein. **b** Portography after the puncture. **c** Portography after the TIPS creation. **d** The short vertical puncture distance (15.44 mm) from Point A to Point B was measured directly with the computer's Picture Archiving and Communication Systems (PACS). Note: The 3D image was made by a 3D visualization planning system (Hokai Company, Zhuhai, China), which is only used to show the position of the hepatic vein and the portal vein and is not used to guide the puncture

All TIPS procedures were performed with the same X-ray angiography system and exposure mode (Philips AlluraXper FD20, Philips, Amsterdam, the Netherlands; Abdomen Frontal: 3 fps, Fluoroscopy: Normal level; Supplemental Fig. 1). LIJ was chosen as first-line access upfront or after a failed TIPS attempt from the RIJ access. A 10-Fr sheath (RUPS-100, Cook, Indiana, USA) was cannulated into the inferior vena cava. A 14-gage Stiffening Cannula (RUPS-100, Cook, Indiana, USA) was introduced into the middle hepatic vein and confirmed via hepatic venography, as shown in Fig. 2. A schematic diagram is shown in Fig. 3. Portal vein puncture was guided with indirect portal vein angiography and performed via the celiac trunk or superior mesenteric artery. The needle punctures, site of portal vein puncture, fluoroscopy time (s), and radiation dose (mGy) were recorded from middle hepatic venography to portal vein puncture success. Then, a balloon angioplasty catheter was inserted and deployed, and covered stents (W.L. Gore & Associates, Flagstaff, AZ, USA) measuring 8 mm in diameter and 50 or 60 mm in length were placed. An example is illustrated in Fig. 4. The portosystemic pressure gradient (PSG) was measured before and after shunt creation. Six operators in our center performed the TIPS procedure with more than five years of experience (Supplemental Table 2). Bedside abdominal ultrasonography was performed to observe whether there was hemoperitoneum. Patients were followed up for 3 months after discharge. The follow-up protocol included assessment of recurrent bleeding, ascitis remission, puncture-related complications, hepatic encephalopathy (HE), routine blood tests, biochemistry, coagulation, color Doppler ultrasound, and enhanced CT of the upper abdomen to check stent patency.

Student's *t*-test was used to compare continuous variables, and categorical variables were compared using the Pearson χ^2^ test. The baseline patient data of the two groups (Supplemental Table 1) were matched with 1:1 propensity score matching (PSM). SPSS Statistics 26.0 (IBM, Armonk, NY) and R software package 3.5.0 (R Foundation for Statistical Computing, Vienna, Austria) were used for statistical analyses. Statistical tests were 2-tailed, and a *P*-value ≤ 0.05 was considered to indicate statistical significance.

**Table 1:** Baseline patient characteristics after propensity score matching

Characteristics	All (*n* = 58)	LIJ-TIPS (*n* = 29)	RIJ-TIPS (*n* = 29)	*P*-value
Sex				1.000
Male	52 (89.7%)	26 (89.7%)	26 (89.7%)	
Female	6 (10.3%)	3 (10.3%)	3 (10.3%)	
Median age (range), years	56.3 [30.0;80.0]	56.6 [30.0;80.0]	55.0 [39.0;80.0]	0.865
Hepatitis B				0.277
Yes	49 (88.0%)	26 (89.7%)	23 (79.3%)	
No	9 (12.0%)	3 (10.3%)	6 (20.7%)	
Child–Pugh class				0.961
A	23 (39.7%)	12 (41.4%)	11 (37.9%)	
B	27 (46.6%)	13 (44.8%)	14 (48.3%)	
C	8 (13.8%)	4 (13.8%)	4 (13.8%)	
Clinical symptoms				0.638
Variceal bleeding	26 (44.8%)	14 (48.3%)	12 (41.4%)	
Refractory ascites	19 (32.8%)	10 (34.5%)	9 (31.0%)	
Variceal bleeding + refractory ascites	13 (22.4%)	5 (17.2%)	8 (27.6%)	
Slices on CT				0.956
3	17 (29.3%)	9 (31.0%)	8 (27.6%)	
4	33 (56.9%)	16 (55.2%)	17 (58.6%)	
5	8 (13.8%)	4 (13.8%)	4 (13.8%)	
Vertical puncture distance on CT, mm ^#^	19.50 ± 0.50	19.10 ± 0.60	19.30 ± 0.60	0.840
Vertical puncture distance on DSA, mm ^#^	22.00 ± 0.58	22.02 ± 0.69	22.23 ± 0.64	0.822

Unless otherwise indicated, data are the number of patients, with percentages in parentheses; ^#^ means ± standard deviation; slices on CT: the number of vertical slices (0.5 cm thick) from the hepatic vein orifice to the puncture point of the portal vein on preoperative CT images. CT, computed tomography. A *P*-value ≤ 0.05 was considered to indicate statistical significance

## Results

The difference in the average vertical puncture distances on CT or DSA between LIJ-TIPS and RIJ-TIPS was not significant (19.10 ± 0.60 mm *vs.* 19.30 ± 0.60 mm, *P* = 0.840; 22.02 ± 0.69 mm *vs.* 22.23 ± 0.64 mm, *P* = 0.822, respectively). There was no significant difference in the baseline characteristics between the two groups after PSM (Table 1).

The average number of needle punctures, fluoroscopy time, and radiation dose in LIJ-TIPS were significantly lower than those in RIJ-TIPS (2.07 ± 0.20 *vs.* 4.10 ± 0.24, *P* < 0.001; 78.45 ± 12.80 s *vs.* 201.16 ± 23.71 s, *P* < 0.001; 31.55 ± 7.04 mGy *vs.* 136.69 ± 16.38 mGy, *P* < 0.001, respectively). Within three punctures, the technical success rate in LIJ-TIPS was significantly higher than that in RIJ-TIPS (86.2% *vs.* 27.6%, *P* < 0.001). There was no significant difference in the mean PSG reduction between LIJ-TIPS and RIJ-TIPS (14.23 ± 1.23 mmHg *vs.* 13.51 ± 0.91 mmHg, *P* = 0.637). During the 3-month follow-up period, a 100% bleeding control rate was achieved in all of the patients. The remission rate of ascites was 73.3% (11/15) in LIJ-TIPS and 70.6% (12/17) in RIJ-TIPS (*P* = 0.863). (Table 2).

**Table 2:** Procedure details and outcomes

Characteristics	LIJ-TIPS (*n* = 29)	RIJ-TIPS (*n* = 29)	*P*-value
Needle punctures ^#^	2.07 ± 0.20	4.10 ± 0.24	< 0.001*
≤ 3, %	25 (86.2%)	8 (27.6%)	< 0.001*
> 3, %	4 (13.8%)	21 (72.4%)	
Fluoroscopy time of puncture, second ^#^	78.45 ± 12.80	201.16 ± 23.71	< 0.001*
Radiation dose of puncture, mGy ^#^	31.55 ± 7.04	136.69 ± 16.38	< 0.001*
Point B			0.525
Left branch of portal vein	16 (55.2%)	13 (44.8%)	
Right branch of portal vein	7 (24.1%)	11 (37.9%)	
Bifurcation of portal vein	6 (20.7%)	5 (17.2%)	
PSG, mmHg ^#^			
Pre-TIPS	25.80 ± 1.28	26.45 ± 1.32	0.725
Post-TIPS	11.56 ± 0.91	12.94 ± 0.92	0.293
Reduction	14.23 ± 1.23	13.51 ± 0.91	0.637
Symptom control			
No rebleeding	19 (100%)	20 (100%)	
Ascites remission	11/15 (73.3%)	12/17 (70.6%)	0.863
Hemoperitoneum			0.038*
Minor	3 (10.3%)	0 (0%)	
Severe	1 (3.4%)	0 (0%)	
Hepatic encephalopathy			0.640
Mild	2 (6.9%)	1 (3.4%)	
Moderate	1 (3.4%)	1 (3.4%)	

Unless otherwise indicated, data are the number of patients, with percentages in parentheses; ^#^ means ± standard deviation; Point B, puncture point of the portal vein; TIPS, transjugular intrahepatic portosystemic shunt; PSG, portosystemic pressure gradient. *A *P*-value ≤ 0.05 was considered to indicate statistical significance

The incidence of hemoperitoneum in LIJ-TIPS was significantly lower than that in RIJ-TIPS (0% *vs.* 13.8%, *P* = 0.038). In RIJ-TIPS, three patients had minor hepatic subcapsular hematoma and recovered after conservative treatment. One patient underwent hepatic arteriography and effective embolization due to severe intra-abdominal bleeding with a 22 g/L drop in hemoglobin within four hours. These patients required an average of up to 5.75 punctures to achieve a successful TIPS. Three patients of LIJ-TIPS and two patients of RIJ-TIPS developed mild/moderate HE, but these symptoms improved through medical treatment. (Table 2).

## Discussion

The LIJ can be a substitute access portal for TIPS, but limited data on its usefulness exist [9, 10]. This single-center retrospective study compared RIJ versus LIJ access for TIPS creation. The LIJ was used in this study as a first-line approach or after failed RIJ access. Average vertical puncture distances of less than 25 mm were achieved in this study. The average needle puncture, fluoroscopy time, radiation dose of X-ray, and hemoperitoneum risk during puncture can be reduced through LIJ access. Therefore, an LIJ-TIPS approach may be more suitable for such patients.

The relative spatial positions of the hepatic and portal veins can be changed by severe shrinkage in a cirrhotic liver [7, 8]. Based on the findings of this study, a schematic diagram of LIJ-TIPS and RIJ-TIPS was made to show the different details of the two approaches. The angle between the inferior vena cava and the middle hepatic vein is larger in LIJ-TIPS, which may be the reason why puncture is more effective [8]. Of note, care should be taken when the guidewire or guide passes through the heart area because of the increased length of the path [9]. The large venous structures of the mediastinum can be stretched when the stiffening cannula of the RUPS-100 passes through the two venous angles (the angle between the LIJ and brachiocephalic vein and the angle between the brachiocephalic vein and superior vena cava). Thus, the stiffening cannula should be guided with the guidewire and sheath under fluoroscopy when it passes through the mediastinal veins.

There are some limitations in our research. First, the middle hepatic vein with left portal vein puncture was preferred for TIPS creation, which is not the standard first-line approach (right hepatic vein to right portal vein) and thus could explain the abnormally high rates of hemoperitoneum. In addition, six different operators performed the TIPS procedures, which could bias group comparisons.

The left internal jugular vein could be used as primary access for TIPS creation in patients with a small liver and short vertical puncture distance.

## Supplementary Information

Below is the link to the electronic supplementary material.

Supplementary file1 (DOCX 330 kb)
