# Supplementary material for: Single-Centre Retrospective Study Using Propensity Score Matching Comparing Left Versus Right Internal Jugular Vein Access for Transjugular Intrahepatic Portosystemic Shunt (TIPS) Creation
Source: Cardiovasc Intervent Radiol. 2022 Jan 1;45(5):563–9. doi: 10.1007/s00270-021-03023-9 (PMC9018633; doi:10.1007/s00270-021-03023-9)
Supplement: Supplementary file 1 — Supplementary file1 (DOCX 330 kb) [file 270_2021_3023_MOESM1_ESM.docx]

**Supplemental Table 1**

Baseline patient characteristics before propensity score matching

| **Characteristics** | **All**  **(n=92)** | **LIJ-TIPS (n=32)** | **RIJ-TIPS (n=60)** | ***P*-value** |
| --- | --- | --- | --- | --- |
| Sex |  |  |  | 0.907 |
| Male | 81 (88.0%) | 28 (87.5%) | 53 (88.3%) |  |
| Female | 11 (12.0%) | 4 (12.5%) | 7 (11.7%) |  |
| Median age (range), years | 55.2 [30.0;84.0] | 56.7[32.0;80.0] | 54.4 [30.0;84.0] | 0.350 |
| Hepatitis B |  |  |  | 0.384 |
| Yes | 73 (79.3%) | 27 (84.4%) | 46 (76.7%) |  |
| No | 19 (20.7%) | 5 (15.6%) | 14 (23.3%) |  |
| Child-Pugh class |  |  |  | 0.075 |
| A | 32 (34.8%) | 15 (46.9%) | 17 (28.3%) |  |
| B | 45 (48.9%) | 13 (40.6%) | 32 (53.3%) |  |
| C | 15 (16.3%) | 4 (12.5%) | 11 (18.3%) |  |
| Clinical symptom |  |  |  | 0.847 |
| Variceal bleeding | 39 (42.4%) | 14 (43.8%) | 25 (41.7%) |  |
| Refractory ascites | 34 (37.0%) | 12 (37.5%) | 22 (36.7%) |  |
| Variceal bleeding+refractory ascites | 19 (20.7%) | 6 (18.8%) | 13 (21.7%) |  |
| Slices on CT |  |  |  | <0.001^*^ |
| 3 | 21 (22.8%) | 10 (31.3%) | 11 (18.3%) |  |
| 4 | 45 (48.9%) | 17 (53.1%) | 28 (46.7%) |  |
| 5 | 17 (18.5%) | 5 (15.6%) | 12 (20.0%) |  |
| 6 | 8 (8.7%) | 0 (0.0%) | 8 (13.3%) |  |
| 7 | 1 (1.1%) | 0 (0.0%) | 1 (1.7%) |  |
| Vertical puncture distance in CT, mm ^#^ | 20.82±0.48 | 19.22±0.60 | 21.67±0.49 | 0.006 ^*^ |
| Vertical puncture distance in DSA, mm ^#^ | 25.32±0.70 | 22.24±0.69 | 26.96±0.96 | <0.001^*^ |

**Notes:** Unless otherwise indicated, data are the number of patients, with percentages in parentheses; ^#^ means±standard deviation; slices on CT: the number of vertical slices (0.5 cm thick) from the starting point of the hepatic vein to the puncture point of the portal vein on preoperative CT images. CT, computed tomography. A *P*-value ≤0.05 was considered to indicate statistical significance.

**Supplemental Table 2**

Compare the professional titles of the two groups of operators

| **Title of operators** | **All**  **(n=58)** | **LIJ-TIPS**  **(n=29)** | **RIJ-TIPS**  **(n=29)** | ***P*-value** |
| --- | --- | --- | --- | --- |
|  |  |  |  | 0.780 |
| Three attending physicians | 39 (67.2%) | 19 (65.5%) | 20 (69.0%) |  |
| Three fellows | 19 (32.8%) | 10 (34.5%) | 9 (31.0%) |  |

**Notes:** Data are the number of patients, with percentages in parentheses. A *P*-value ≤0.05 was considered to indicate statistical significance.

**Supplemental Figure 1**


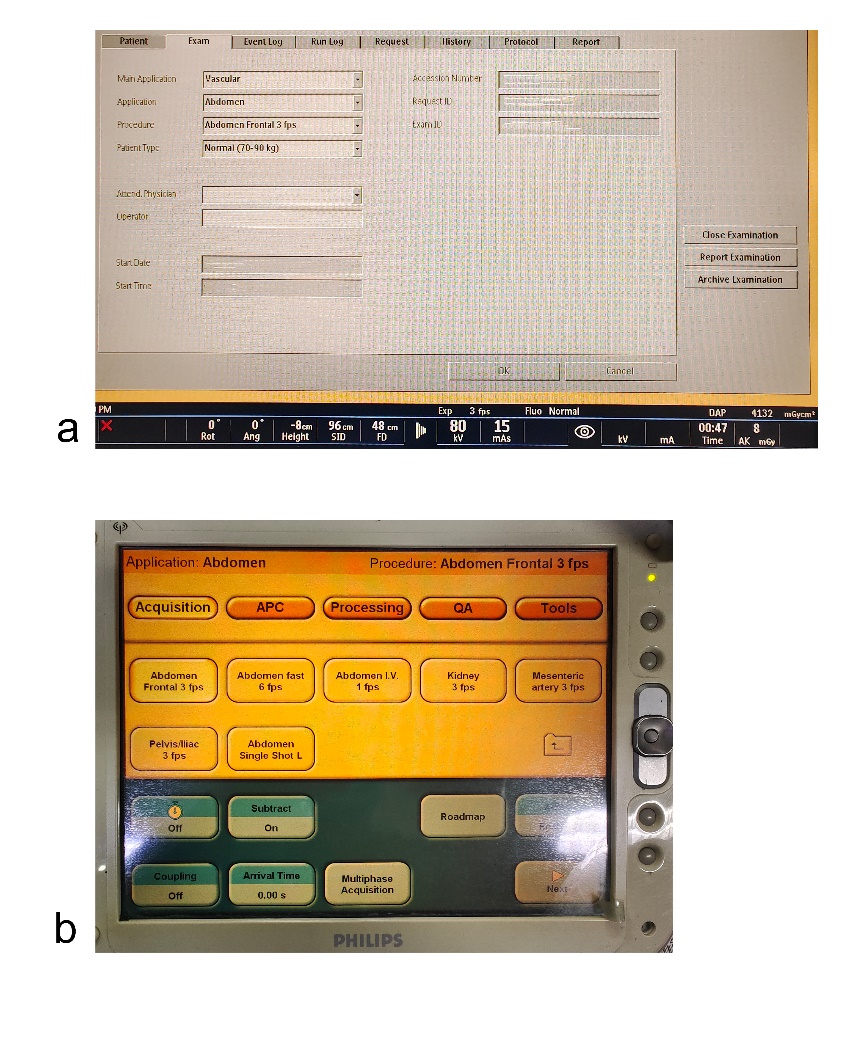


The radiation dose is displayed in the computer controlling DSA. (a) All TIPS procedures were performed with the same X-ray angiography system and exposure mode (Philips AlluraXper FD20, Philips, Amsterdam, the Netherlands; Abdomen Frontal: 3 fps, Fluoroscopy: Normal level). The fluoroscopy time (s) and radiation dose (mGy) were recorded from middle hepatic venography to portal vein puncture success. (b) Console for DSA operating mode.
